# Management of Liddle Syndrome in Pregnancy: A Case Report and Literature Review

**DOI:** 10.1155/2017/6279460

**Published:** 2017-03-15

**Authors:** Michael Awadalla, Manasi Patwardhan, Adham Alsamsam, Nashat Imran

**Affiliations:** ^1^Department of Obstetrics and Gynecology, Wayne State University, Detroit Medical Center, Detroit, MI, USA; ^2^Division of Nephrology and Hypertension, Department of Internal Medicine, Wayne State University, Detroit Medical Center, Detroit, MI, USA

## Abstract

Liddle syndrome is an autosomal dominant genetic condition that causes hypertension and hypokalemia due to a gain-of-function mutation in the SCNN1B or SCNN1G genes which code for the epithelial sodium channel in the kidney. This leads to increased sodium and water reabsorption causing hypertension. We report a case of a 27-year-old pregnant woman who was admitted for hypertension and hypokalemia and later diagnosed and treated for Liddle syndrome using amiloride. Maintaining a high suspicion of Liddle syndrome in pregnancy is essential in such cases to be able to adequately and effectively treat the hypertension. Due to physiological effects of pregnancy, the dose of amiloride may need to be increased as gestational age progresses up to a maximum dose of 30 mg orally per day.

## 1. Background

Liddle syndrome was first described in 1963 by Liddle et al. [1] and is caused by a rare autosomal dominant gain-of-function mutation in one of the genes for the epithelial sodium channel (ENaC) found in the distal convoluted tubule of the kidney nephron. This mutation results in pathological reabsorption of sodium and water from the tubule and excretion of potassium into the urine which results in hypertension, hypokalemia, metabolic alkalosis, and suppression of renin and aldosterone. The ENaC, sometimes referred to as the amiloride-sensitive epithelial sodium channel, is composed of three homologous subunits: alpha (encoded by the SCNN1A gene), beta (SCNN1B gene), and gamma (SCNN1G gene). Mutations in the beta or gamma subunits have been shown to cause Liddle syndrome (Staub, Warnock).

Liddle syndrome often presents with the onset of hypertension at a young age, hypokalemia, decreased renin and aldosterone, and a family history consistent with autosomal dominant inheritance. The phenotypic presentation of Liddle syndrome varies somewhat even within families carrying the same mutation and thus hypokalemia is not always present and the degree of hypertension is variable [2, 3]. Inhibitors of the ENaC that are used in the treatment for Liddle syndrome are, namely, two medications: amiloride (preferred in pregnancy, the Food and Drug Administration classifies it as pregnancy category B) and triamterene (generally avoided in pregnancy due to interference with folic acid metabolism, pregnancy category C). Some patients with Liddle syndrome may respond to only triamterene or amiloride depending on their genetic mutation (Warnock). Treatment with these medications typically results in resolution of the hypertension and hypokalemia.

## 2. Case

**Figure 1:**
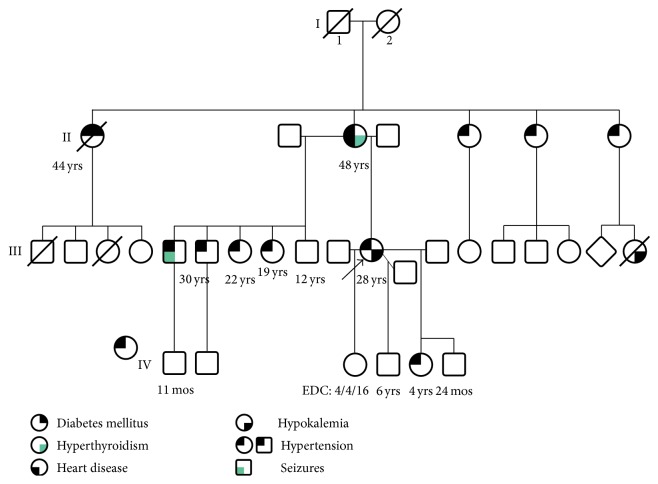
Patient's family pedigree.

A 27-year-old African American woman, gravida 4, para 2-1-0-3, at 18-week gestation with a history of chronic hypertension with no prenatal care presented to triage with conjunctivitis and was found to have a blood pressure of 183/108 mmHg. She had been diagnosed with chronic hypertension at the age of 13 and had multiple partial workups for her hypertension and several hospital admissions for severely elevated blood pressures. Upon review of her medical records, her hypokalemia dated to the age of 17 at least. She had uncomplicated vaginal deliveries in 2008 and 2010. In 2013, she had an induction of labor and vaginal delivery at 28 weeks for severely elevated blood pressure readings up to 250/120 mmHg. She reported a family history of hypertension in her two brothers, two sisters, her mother, and her 4-year old child. She reported one sibling that was unaffected by hypertension. She reported that most of her family members had onset of their hypertension between the ages of 15 and 30 (Figure 1).

On admission, her serum potassium was 2.6 mMol/L, sodium 140 mMol/L, and creatinine 0.47 mg/dL. Her urine potassium was 15 mMol/L, urine sodium was 112 mMol/L, and urine chloride was 110 mMol/L. She had very low renin and aldosterone which is consistent with Liddle syndrome. Her renin activity was 0.8 ng/mL/hr, aldosterone was <3.0 ng/dL, and Aldosterone/Renin Activity (A/RA) was <3.8. The A/RA ratio was difficult to interpret as the renin level was near the lower limit of the reported range and the aldosterone level was below the reported range. Her serum adrenocorticotropic hormone (ACTH), urine metanephrines, and urine vanillylmandelic acid (VMA) were all within normal range. Her baseline 24 hr urine showed 357 mg of protein. A renal US showed normal echotexture and there was no evidence of hydronephrosis or nephrolithiasis. There was no size discrepancy between the two kidneys. A previous computed tomography scan of the abdomen showed normal appearing kidneys, renal vasculature, and adrenal glands. Her extensive laboratory workup and family history were consistent with the provisional diagnosis of Liddle syndrome as other causes were less likely. To confirm her diagnosis, genetic testing for our patient was sent and confirmed a heterozygous frameshift mutation in the SCNN1B gene (Monogenic Hypertension Evaluation, Athena Diagnostics). The patient was notified multiple times to bring her family members in for evaluation and genetic testing but declined to do so.

Initially, she was admitted and her acute hypertension was treated with intravenous hydralazine followed by oral sustained-release nifedipine 30 mg daily and oral labetalol 300 mg twice daily. After her laboratory workup and the provisional diagnosis of Liddle syndrome, she was started on oral amiloride 5 mg daily as an outpatient, which was increased to 5 mg twice daily at 22 weeks of gestation and later to 10 mg in the morning and 5 mg at bedtime. With this amiloride dosing regimen, her serum potassium increased to 3.8 mMol/L without requiring any oral potassium supplementation, but her blood pressure control was still suboptimal. Amiloride was further increased to 10 mg twice daily. Her BP at 24 weeks of gestation was 155/102 mmHg. Unfortunately, the patient was poorly compliant with her amiloride and did not present for any further prenatal care before delivery.

At 37 weeks and 4 days of gestation, she presented to triage in labor and was found to be dilated at 8 centimeters. She had not been taking her amiloride and her blood pressure readings were 186/133 and 212/129 mmHg. Her potassium level was 3.9 mMol/L. Her acute hypertension was controlled with intravenous labetalol. The patient underwent an emergent primary low transverse cesarean section for FHR deceleration. A female infant was born with 1-minute and 5-minute Apgar scores of 8 and 9, respectively, and a weight of 2070 grams (<1% percentile). The umbilical cord arterial pH was 7.20 and the base excess was −6.2. The placental pathology was unremarkable. Postpartum hypertension was treated with oral triamterene 50 mg daily (it was more readily available than amiloride as it was on hospital formulary) and oral labetalol 300 mg three times daily, resulting in BPs in the range of 140–150 s/70–80 s mmHg. She was discharged 3 days after her delivery. She did not follow up for a postoperative or postpartum visit despite frequent reminders. She was treated in the emergency room for a yeast infection 3 months after delivery at which time her blood pressure was 173/111 mmHg, likely secondary to noncompliance with triamterene.

## 3. Discussion

Amiloride appears to be a safe and effective medication for controlling blood pressure and correcting hypokalemia in pregnant patients with Liddle syndrome. A previous case report of Liddle syndrome diagnosed before pregnancy describes the achievement of optimal blood pressure control with oral amiloride 15 mg daily; however, a cesarean delivery was later performed for uncontrolled hypertension in labor (Caretto). Our patient had a c.1789_1790 base pair duplication of cytosine in codon 597 of the SCNN1B gene (heterozygous frameshift mutation). This is one of the different mutations that can lead to the Liddle syndrome phenotype (Jackson).

Diuretics in pregnancy are generally avoided due to possible volume depletion and uteroplacental hypoperfusion. This is counterintuitive to the presumption of volume expansion in Liddle syndrome. The clinical presentation does not usually include edema. Liddle syndrome patients behave clinically similar to patients with primary hyperaldosteronism due to the function of their mutation. In primary hyperaldosteronism, it is known that there is initially sodium and water retention that is followed by a spontaneous diuresis called aldosterone escape which lowers the extracellular fluid volume almost toward normal. This occurs due to volume expansion. The mechanisms responsible for the escape include the following: increased secretion of atrial natriuretic peptide ANP, decreased thiazide-sensitive sodium chloride (NaCl) cotransporter in the distal tubule, and pressure natriuresis. It is possible that the same autodiuresis may be involved in Liddle syndrome, and hence diuretics should be avoided [4].

Amiloride is an effective medication in the treatment of Liddle syndrome in pregnancy since it reverses the pathological volume expansion and the excessive sodium reabsorption, thus, mitigating the pathogenesis of hypertension. In rats, the amount of alpha ENaC subunits, the rate-limiting subunit for formation of the ENaC channel, has been shown to increase in late pregnancy (West) which is consistent with an increase in the number of ENaC channels as pregnancy progresses. This is consistent with our clinical experience and that of Caretto's group [5] that increasing doses of amiloride are needed to control BP as gestational age advances due to the presence of more pathological ENaC channels. Amiloride doses of up to 15 mg orally twice a day have been used during pregnancy (Mathen). Titrating amiloride to a target blood pressure of 140–150 s/90–100 s mmHg seems reasonable with a maximum dose of 30 mg daily. Warnock has suggested that, in patients with Liddle syndrome who correct their potassium with ENaC-blocking medication but remain hypertensive, the addition of beta blockers or vasodilators may be beneficial [6]. By the third day after delivery, the blood pressure of the patient described by Caretto et al. had normalized to 125/85 mmHg [5]. In contrast, the blood pressure of the patient in our case remained elevated after delivery, which could be likely due to noncompliance with triamterene. Due to the potential for the number of ENaC channels to change after delivery, the blood pressure medication regimen should be reevaluated after a patient with Liddle syndrome delivers. The safety of both amiloride and triamterene in lactation is unknown; hence close monitoring of the breastfed infant is advised. Pregnant patients with Liddle syndrome may require additional agents to achieve their blood pressure target which could be done by adding a second agent to the first-line medications, the ENaC blockers. There is limited literature on this topic; however, it is well accepted that a poorly controlled blood pressure in pregnancy is a major risk factor for adverse prenatal outcome and hence more medications may be needed [7].

Although only a few cases of Liddle syndrome in pregnancy have been reported, there is an association with both fetal growth restriction and preeclampsia. One case described by Caretto et al. was complicated by fetal growth restriction without preeclampsia or proteinuria [5]. Hayes et al. reported on the anesthetic management of a cesarean hysterectomy for a patient with Liddle syndrome and placenta accreta in her third pregnancy [8]. The case report notes that the woman's two previous deliveries were complicated by fetal growth restriction and preeclampsia. The patient's third pregnancy was complicated by fetal growth restriction but not preeclampsia. Our case was associated with fetal growth restriction less than the 1st percentile at delivery. Our patient had 357 mg protein in a baseline 24 hr urine sample at 18 weeks of gestation and did not develop increased proteinuria in her pregnancy (her urinalysis on the day of delivery was negative for protein). Although diagnosis of preeclampsia in patients with Liddle syndrome can be challenging, standard criteria for diagnosis of chronic hypertension with superimposed preeclampsia should be used.

In summary, a high suspicion for Liddle syndrome in pregnancy should be maintained in cases with uncontrolled hypertension and hypokalemia with a significant family history of the same. Management should involve controlling the acute severe elevation of blood pressure with intravenous hydralazine or labetalol as per standard obstetric protocols, replacing potassium and initiating the oral ENaC blocker (amiloride during pregnancy and either amiloride or triamterene afterward). Due to a 2-month turnaround time for genetic testing, empiric treatment with amiloride is reasonable. Weekly prenatal care appointments should be scheduled during initiation of amiloride to monitor blood pressure and potassium and to titrate the dose along with evaluation of preeclampsia and fetal growth restriction. Finally, encourage first-degree relatives to be evaluated for elevated blood pressure and hypokalemia, including the newborn infant.

## Additional Points

(1) Until genetic testing results are available, suspected Liddle syndrome can be diagnosed and treated empirically based on onset of hypertension at a young age often less than 20 years old, hypokalemia, decreased renin and aldosterone, and a family history consistent with autosomal dominant inheritance. (2) In pregnancy, a reasonable starting dose of oral amiloride is 5 to 10 mg total daily as once-a-day dosing or divided for twice-a-day dosing. As gestational age progresses, higher doses of amiloride up to 30 mg daily divided into two doses may be required. It seems reasonable to titrate amiloride to a target blood pressure range of 140–150 s/90–100 s and a potassium greater than 3.5. (3) Amiloride is preferred in pregnancy over triamterene for treatment of Liddle syndrome due to its safety profile in pregnancy.
